# Chronic heart failure health-related quality of life questionnaire (CHFQOLQ-20): development and psychometric properties

**DOI:** 10.1186/s12872-023-03197-9

**Published:** 2023-03-29

**Authors:** Abdoljavad Khajavi, Mahdi Moshki, Shima Minaee, Farveh Vakilian, Ali Montazeri, Haydeh Hashemizadeh

**Affiliations:** 1 Department of Community Medicine, School of Medicine, Gonabad University of Medical Sciences, Gonabad, Iran; 2 Department of Health Education and Health Promotion, School of Health, Gonabad University of Medical Sciences, Gonabad, Iran; 3 Social Development and Health Promotion Research Center, Gonabad University of Medical Sciences, Gonabad, Iran; 4 Department of Cardiovascular Diseases, Razavi Hospital, Mashhad, Iran; 5 Department of Cardiology, Preventive Atherosclerotic Research Center, Imam Reza Hospital, Faculty of Medicine, Mashhad University of Medical Sciences, Mashhad, Iran; 6 Health Metrics Research Center, Iranian Institute for Health Sciences Research, ACECR, Tehran, Iran; 7 Faculty of Humanity Sciences, University of Sciences & Culture, Tehran, Iran; 8 Department of Nursing, Quchan Branch, Islamic Azad University, Quchan, Iran

**Keywords:** Patient-reported outcome measure, Health-related quality of life, Chronic heart failure, Reliability, Validity, Iran

## Abstract

**Background:**

Health-related quality of life (HRQoL) is an important outcome indicator for chronic diseases. This study aimed to develop a new instrument for assessment of HRQoL in chronic heart failure (CHF) and evaluate its psychometric properties.

**Methods:**

This study included two steps of conceptualization and item generation, and assessment of the psychometric properties of an instrument for measuring HRQoL in patients with CHF. A sample of 495 patients with confirmed diagnosis of heart failure participated in the study. In addition to content validity, exploratory and confirmatory factor analyses, concurrent validity, convergent validity, known groups comparison were performed to assess construct validity. Internal consistency, and stability were estimated by the Cronbach’s alpha, the MacDonal’s Omega, and intraclass correlation coefficients.

**Results:**

The content validity of the developed chronic heart failure quality of life questionnaire was assessed by 10 experts. The exploratory factor analysis indicated a four-factor solution for the instrument containing 21 items that jointly accounted for 65.65% of variance observed. The confirmatory factor analysis confirmed the four factor solution with the following fit indexes (χ^2^/df = 2.214, CFI = 0.947, NFI = 0.91, TLI = 0.937, IFI = 0.947, GFI = 0.899, AGFI = 0.869, RMSEA = 0.063). However, at this stage one item was removed. The concurrent and convergent validity of the CHFQOLQ-20 were established using the Short Form Health Survey (SF-36), and the MacNew Heart Disease Quality of Life Questionnaire, respectively. The known-groups validity as assessed by using the New York Heart Association (NYHA) functional classification showed that the questionnaire discriminated well between patients who differed in functional classification. The internal consistency and test-retest reliability of the CHFQOLQ-20 were satisfactory, with a Cronbach’s alpha and intraclass correlation coefficient (ICC) values of 0.93 and 0.84, respectively.

**Conclusion:**

The results confirmed that CHFQOLQ-20 is a valid and reliable instrument for measuring quality of life (QoL) in patients with CHF. It is a short and easy-to-use instrument that is also capable of assessing the cognitive functioning, which has been overlooked in previous questionnaires.

## Introduction

Heart failure (HF) is an important public health problem in different communities worldwide. Approximately 64.3 million people worldwide suffer from HF, and its prevalence in developed countries is 1–2% of the general population of adults [1]. HF is a chronic progressive disease with a significant symptom burden, impaired quality of life (QoL), and high rate of mortality [2]. The QoL of patients with HF is lower than the QoL of the general population and patients with other chronic diseases. Patients often experience a number of physical and psychological symptoms such as dyspnea, fatigue, edema, sleep disorders, chest pain, and depression. Thus, their physical and social activities are impaired, and their health-related QoL (HRQoL) decreases [3]. The prognosis of HF is poorer than many cancer types [4].

At present, patient-reported outcome measurement is a valuable tool for assessment of the daily activities and limitations in daily living and QoL of patients [5]. Accordingly, questionnaires are the only tools for assessment of HRQOL and the effects of HF on daily life and activities of patients [6]. HRQOL is an important outcome indicator in chronic diseases because the conventional indicators (such as physical, physiological, and biochemical indicators) cannot comprehensively assess the effect of disease or treatment on patients. Thus, assessment of HRQoL is important for monitoring and follow-up of patients [7]. Patients with heart failure often experience a cognitive decline following impairment of their hemodynamics, and cerebral hypo-perfusion [8]. Several qualitative studies on the perspective of patients have reported cognitive disorders as adverse effects of HF on QoL [9–12]; however, none of the available instruments have a cognitive disorder domain [13]. In a holistic assessment of health, it is important to include subjective cognitive wellbeing, as a component of HRQoL in HF, in order to precisely assess the memory, clarity of thoughts, and mental ability of patients in their important daily tasks, leisure activities, self-care, safety, and adherence to medication intake [14].

The HRQoL of patients with HF can be assessed by several generic and disease-specific instruments. The generic measures include sickness impact profile (SIP) [15] and the 36-item Short Form Health Survey (SF-36) [16] that assess the health status, as well as the disease-specific instruments for HF which are more sensitive to clinical changes and include Quality of Life Questionnaire in Severe Heart Failure (QLQ-SHF) [17], Chronic Heart Failure Questionnaire (CHFQ) [18], Left Ventricular Disease Questionnaire (LVDQ) [19], the Kansas City Cardiomyopathy Questionnaire (KCCQ) [20], the Minnesota Living With Heart Failure Questionnaire (MLHFQ) [21], and the Chronic Heart Failure Assessment Tool (CHAT) [4]. However, some of the available tools for this purpose have methodological limitations. For instance, CHFQ [18] and LVDQ [19] have a small sample size. Also, the structural validity of patient reported outcome measurement instruments is evaluated by exploratory factor analysis (EFA) and confirmatory factor analysis (CFA); while, assessment of the underlying factors by CFA has not been performed in some of them such as CHAT [22], or is not clear in some others. The MLHFQ is among the most commonly used measures. Several investigators such as the developers of MLHFQ have assessed the psychometric properties of this instrument; however, some concerns still exist regarding the homogeneity of some items and their validity. Heo in her PhD thesis assessed the psychometric properties of the MLHFQ in a larger sample size (638 patients) and showed that seven items of this questionnaire may not properly reflect the common concerns of the majority of patients. Also, 16 out of 21 items of the MLHFQ were loaded on three factors, and similar to the results of previous analyses, only two factors (physical and emotional) were meaningful clusters. Furthermore, the physical scale of MLHFQ moderately assesses the physical measures, and its emotional scale poorly evaluates this domain [23].

The significance of assessment of the problems of patients with HF and their treatment outcomes highlights the need for developing a valid, reliable, and responsive tool for this purpose. HRQoL is influenced by the experiences, beliefs, expectations, and perceptions of individuals, and reflects the cognitive perception of individuals from the effects of HF on their life; whereas, the majority of the available tools for this purpose are expert-driven rather than patient-driven [22]. The majority of HRQoL tools for patients with HF have been designed in Europe and North America; while, significant cultural and social differences exist between the eastern and western worlds which can affect the QoL. In an international consensus process comprising of experts in psychology, epidemiology, statistics, and clinical medicine from all over the world, a consensus was reached regarding the taxonomy, definitions of measurement properties, and measurement properties for health-related patient-reported outcomes. Accordingly, consensus-based standards for the selection of health measurement instruments (COSMIN) checklist was designed [24, 25]. In most cases, patient-reported outcome measures face a great challenge due to lack of compatibility with the standard systems and frameworks. The most comprehensive and widespread attempt of the World Health Organization (WHO) in this respect was to create a standard framework entitled International Classification of Functioning, Disability and Health (ICF). This framework is a comprehensive system according to a physiological, biological and social model, which can be adapted to many health measurement and QoL assessment tools. Concepts such as QoL, health, and function are supported by this framework [26]. Since the methodology of none of the HF measurement tools has been in accord with the COSMIN panel with the application of ICF, this study aimed to develop an instrument for assessment of HRQoL in chronic heart failure (CHF) and assess its validity and reliability from the perspective of Iranian patients according to the COSMIN recommendations on terminology and research agenda with ICF application for maximum optimization of this instrument.

## Methods

### Participants

As explained in the following section we recruited different samples for the study. For the first step in addition to ten experts, ten patients with heart failure participated in the study. For the second step we recruited a connivance sample of patients with heart failure. Although updated information does not exist on heart failure statistics, a hospital-based study of cardiology wards across Iran in 2012 reported that the incidence rate of HF was 8.1%. The study also reported that the HF incidence was higher in women than that of men (8.6% vs. 7.9%, respectively) [27]. However, the statistical population for this study consisted of all patients with heart failure attending the outpatient clinics of a teaching hospital affiliated to Mashhad University of Medical Sciences., Mashhad, Iran during March 2018 to January 2019. An HF specialist screened the patients with respect to the eligibility criteria using the New York Heart Association functional classification (NYHA) [28]. Participants with confirmed diagnosis of CHF, and ejection fraction ≤ 40% were enrolled. Patients with cognitive disorders were excluded.

### Procedure

The study was conducted in two steps: (1) item generation (2) psychometric study.

1. Item generation: Items were generated by performing a literature review, and conducting qualitative studies [29, 30]. An extensive review of the literature was conducted with special emphasis on theories, models, and existing instruments for measuring health-related quality of life in patients with CHF. We compared the content of the existing instruments based on the global framework for the comparison of HRQOL measurement instruments based on the World Health Organization’s guideline entitled International Classification of Functioning, Disability and Health (ICF) [4]. The qualitative study addressed quality of life related issues from the perspective of 19 selected patients with heart failure through semi-structured interviews and framework analysis approach. The participants consisted of 6 females and 13 males, with the mean age of 59.5 ± 12.4 years, the mean disease duration of 5.2 years, and the mean ejection fraction of 23.1 ± 8.3. Accordingly, 37 items were generated. Response categories ranged from 5 = not at all, to 1 = very much) with regard to the effect of heart failure on daily life of patients within the past 4 weeks. Then, 10 experts including HF fellowship cardiologists, HF specialized nurses, and psychologists evaluated the initial 37-items according to Waltz and Bausell content validity index (CVI). The experts scored the relevance, clarity, and simplicity of each item using a four-point Likert scale. The CVI of each item was calculated by dividing the number of experts that gave a score of 3 or 4 to a particular item by the total number of experts [31]. Items with a CVI ≥ 0.79 were accepted. The mean CVI scale was 0.91 (0.87-1) and thus at this stage all items kept. The necessity of each item was analyzed using a three-point rating scale of (I) not necessary, (II) useful, but not essential, and (III) essential. Following assessment by the experts, the content validity ratio (CVR) for each item was calculated. According to the Lawshe’s table, the acceptable CVR for 10 experts was set at 0.62. In assessment of CVR, six items were omitted according to the cut-off point. Thus, the 37-item questionnaire was changed to a 31-items questionnaire. Consequently, we performed face validity. Ten patients with HF completed the questionnaire and they were asked to indicate the importance of each item on a 5-point Likert scale. As such the impact score was calculated and a cut-off point of 1.5 was thought satisfactory. Since the impact score for two items were below 1.5, these two items were removed and the questionnaire with 29 items was subjected to psychometric assessments.
2. Psychometric study: A cross sectional study was conducted on a sample of patients with chronic heart failure from March 2018 to January 2019. Participants completed the questionnaire while they were briefed about the questionnaire and signed informed consent form. Patients were ensured that they were free to quit whenever they wished to do so. In addition, they have completed the following questionnaires.

#### The short Form Health Survey (SF-36)

It was used for assessment of concurrent validity. The SF-36 is a well-known generic HRQOL measure with eight subscales of physical functioning, role limitations due to physical problems, role limitations due to emotional problems, vitality, mental health, social functioning, bodily pain, and general health perception. Scores on each item range from 0 to 100 where a higher score indicates higher HRQoL. The psychometric properties of the Iranian version of SF-36 are well documented. The Cronbach’s alpha coefficients ranged from 0.77 to 0.90 with the exception of the vitality subscale (alpha = 0.65) [32]. Since it was expected that the disease-specific HRQOL would be moderately correlated with a generic HRQOL instruments, we hypothesized that the CHFQOLQ-20 would have a moderate positive correlation with the SF-36.

#### The MacNew Heart Disease Quality of Life Questionnaire

It is a disease-specific questionnaire and was used for assessment of convergent validity. It was designed for myocardial infarction patients. Moreover, it has been acceptably used for patients with HF as well. It has 27 questions and three domains namely physical, psychological, and social. Higher scores indicate higher QoL. The psychometric properties of the Iranian version of this questionnaire are well reported. Internal consistency of the Iranian version as estimated by the Cronbach’s alpha coefficient ranged from 0.92 to 0.95 [33], and its validity and reliability for patients with CHF have been confirmed [34]. The Cronbach’s alpha for this questionnaire was 0.95. It was hypothesized that a moderate positive correlation between the CHFQOLQ-20 and the MacNew questionnaire would be achieved.

#### The New York Heart Association (NYHA) functional classification

It is a clinical classification ranging from 1 to 4 which is extensively used for assessment of cardiac functional capacity [28]. The NYHA class was determined by a specialist who had a fellowship in HF. It was used for known groups comparison and hypothesized that those with higher scores on the CHFQOLQ-20 would have a better functioning as measured by the NYHA functional class.

### Data analysis

Descriptive statistics were applied to summarize the clinical and demographic characteristics of patients. The exploratory factor analysis (EFA) was performed to explore the items and their underlying structure using principal component analysis by oblique rotation. To extract the number of factors, the scree plot, Eigenvalues greater than 1, and parallel analysis were applied. To justify undertaking a factor analysis, the Barrette test of sphericity was used. The factor loading threshold was considered ≥ 0.4. To fit the underlying structure with the observed data, the confirmatory factor analysis (CFA) with the maximum likelihood method was used. The goodness-of-fit indices to confirm the model fit included the χ^2^/df ratio < 3, comparative fit index (CFI) > 0.95, normed fit index (NFI) > 0.9, goodness-of-fit index (GFI) > 0.9, adjusted GFI (AGFI) > 0.9, and root-mean-square error of approximation (RMSEA) < 0.08 [35, 36]. To assess the concurrent and convergent validity, the Pearson’s correlation coefficient was used. In addition, convergent and discriminated validity were assessed by obtaining the average variance extracted (AVE) which should be equal to or greater than 0.5, while discriminant validity was established if the AVE was greater than maximum shared squared variance (MSV), and average shared squared variance (ASV) [37].

To assess known-groups comparison, ANOVA was applied. Furthermore, scaling assumption was assessed by performing item-scale correlation. It was hypothesized that items belonging to given factors would have higher correlation with its own corresponding factor than other factors. The reliability of CHFQOLQ-20 was assessed by internal consistency (Cronbach’s alpha), MacDonald’s Omega, and test-retest analysis (intraclass correlation coefficient-ICC). The internal consistency (Cronbach’s alpha) equal or greater than 0.7 was considered to be acceptable. Fifty patients who had no change in their treatment protocol, and not received any intervention affecting their QoL were selected for test-retest with a 2-week interval, and the ICC value of > 0.7 was considered acceptable for test-retest reliability assessment. The SPSS version 25 was used for the EFA, and the SPSS Amos was used for the CFA.

### Sample adequacy and power analysis

Overall 495 patients were included in the study. Of these, information for 160 patients used for exploratory factor analysis and the data from the remaining 335 patients were used for the confirmatory factory analysis (Table 1). All other analyses were performed using the information from all participants. The adequacy of sample size for the exploratory factory analysis was checked by the Kaiser-Meyer-Olkin test and the “power analysis” for the confirmatory factor analysis was performed by using Danial Soper’s online Free-Software (available at: https://www.danielsoper.com/statcalc/calculator.aspx?id=89).

**Table 1:** The characteristics of participants

	EFA sample (n = 160)	CFA sample (n = 335)
Age, year (mean, SD)	60.0 (11.1)	59.4 (12.7)
Gender, male (no., %)	112 (70)	228 (68)
Living with partner/spouse (no., %)	147 (91.8)	302 (90.1)
Years of education (mean, SD)	7.5 (3.1)	8.9 ( 4.4)
Body mass index (kg/m^2^)	24 (3.9)	26 (4.9)
**Heart failure characteristics**		
Ischemic heart failure, (no., %)	116 (72.5)	239 (71.3)
Duration since heart failure, years (mean, SD)	4.9 (1.9)	(2.1) 5
Left ventricular ejection fraction (no., %)	24.9 (0.9)	26.6 ( 0.7)
Hypertension (no., %)	44 (27.5)	97(28.9)
CABG (no., %)	54 (33.7)	120 (35.8)
Diabetes (no., %)	63 (39.3)	147 (43.8)

In this regard with alpha set at 0.05 (two-tailed), a factor analysis with one latent variable (heart failure quality of life) and four observed variables (derived from exploratory factor analysis), statistical power set at 0.9, and a small effect size of 0.1 (according to the effect size conventions in Daniel Soper’s website), 199 participants were regarded as minimum sample size required.

## Results

### Exploratory factor analysis

**Fig. 1:**
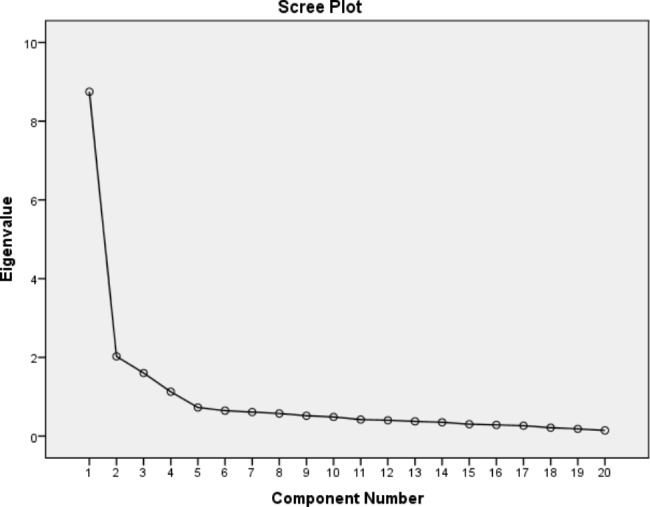
Scree plot for the CHFQOLQ-20 (n = 160)

The initial results obtained from EFA showed low loading (< 0.3) for 8 items. Accordingly, the number of items was reduced to 21 and once more the underlying factor structure was assessed. The result of Kaiser-Meyer-Olkin test was 0.925, indicating that factor analysis can be used to determine the dimensions. The result of Barrette test of sphericity was also statistically significant as follows: χ^2^ = 4054.903, df = 190, P ≤ 0.001. Both tests showed sample adequacy for the conduction of EFA. The criteria used to determine the number of factors included eigenvalue > 1, Scree plot and factor loading of > 0.4, and the theoretical considerations. The initial EFA extracted a four-factor solution. The scree plot (Fig. 1) suggested 2 to 5 factors, and eventually, by the parallel analysis (by using the Monte Carlo software) four factors were extracted that explained 65.65% of the total variance observed. Since the framework used for this study was ICF, the factors were labeled by the ICF underlying construct. The factor loading of each item was higher than 0.5 except for item 9 which had a factor loading of 0.446. The first factor extracted by EFA was labeled as ‘physical functioning’. This factor contained 10 items, which explained a variance of 42.016% observed. The second factor was ‘cognitive functioning’ with four items, that explained 9.906% of variance observed. The third factor was named as ‘general health’. It contained three items that explained 7.705% of variance. The fourth factor was labeled as ‘mental health’. It had four items which explained a variance of 6.025% observed (Table 2).

**Table 2:** Results obtained from exploratory factor analysis for the CHFQOLQ (n = 160)

	Items	Factor 1 (Physical functioning)	Factor 2 (Cognitive functioning)	Factor 3 (General health)	Factor 4 (Mental health)
	**Due to chronic heart failure, in the past 4 weeks**:				
1	Have you had trouble walking around the house?	**0.789**	0.000	-0.073	0.126
2	Did you have trouble taking a short walk (one block or around 100 m)?	**0.888**	-0.079	-0.032	0.084
3	Did you experience difficulty in doing house chores (such as gardening, moving things, vacuum cleaning, or daily grocery shopping)?	**0.894**	-0.052	-0.022	0.030
4	Did you have trouble climbing up the stairs one floor with no rest?	**0.9**	0.006	0.014	-0.047
5	Did you have trouble fast walking for a few blocks or over 100 m?	**0.876**	-0.041	0.041	-0.091
6	Did you experience difficulty in lifting heavy things (such as furniture or suitcase)?	**0.838**	-0.010	-0.043	-0.115
7	Did you have to lie-down or sit down during the day?	**0.757**	0.132	0.005	-0.010
8	Did you experience trouble in exercising, leisure activities or going on a trip?	**0.630**	0.088	0.114	0.107
9	Did you feel fatigue and low energy level?	**0.464**	0.163	0.159	0.155
10	Did you experience dyspnea during physical activities?	**0.608**	0.071	0.183	-0.023
11	Did you have problem concentrating (e.g. concentration in reading a topic or watching TV)?	0.087	**0.752**	-0.011	0.062
12	Did you have problem remembering things related to a few days earlier (e.g. remembering the location of things or an appointment)?	-0.053	**0.899**	0.045	-0.104
13	Did you have limitation in learning new topics or skills?	-0.017	**0.875**	0.035	0.050
14	Did you have problem in making decisions?	0.087	**0.522**	-0.142	0.423
15	How do you estimate your current general health status?	-0.019	-0.015	**0.867**	0.072
16	How do you assess your current health status in comparison with the past year?	0.046	-0.024	**0.879**	-0.088
17	How do you estimate your current quality of life?	-0.008	0.060	**0.742**	0.091
18	Do you feel as you are a burden to others?	0.360	0.035	0.038	**0.501**
19	Do you feel sad and depressed?	0.131	0.080	0.142	**0.569**
20	Do you feel useless and worthless?	0.083	-0.041	0.145	**0.735**
21	Do you have self-confidence*	-0.120	-0.016	-0.027	**0.757**
	*Eigen values*	8.823	2.080	1.618	1.265
	*Variance (%)*	42.016	9.906	7.705	6.025

This item was removed after confirmatory factor analysis

### Confirmatory factor analysis

**Fig. 2:**
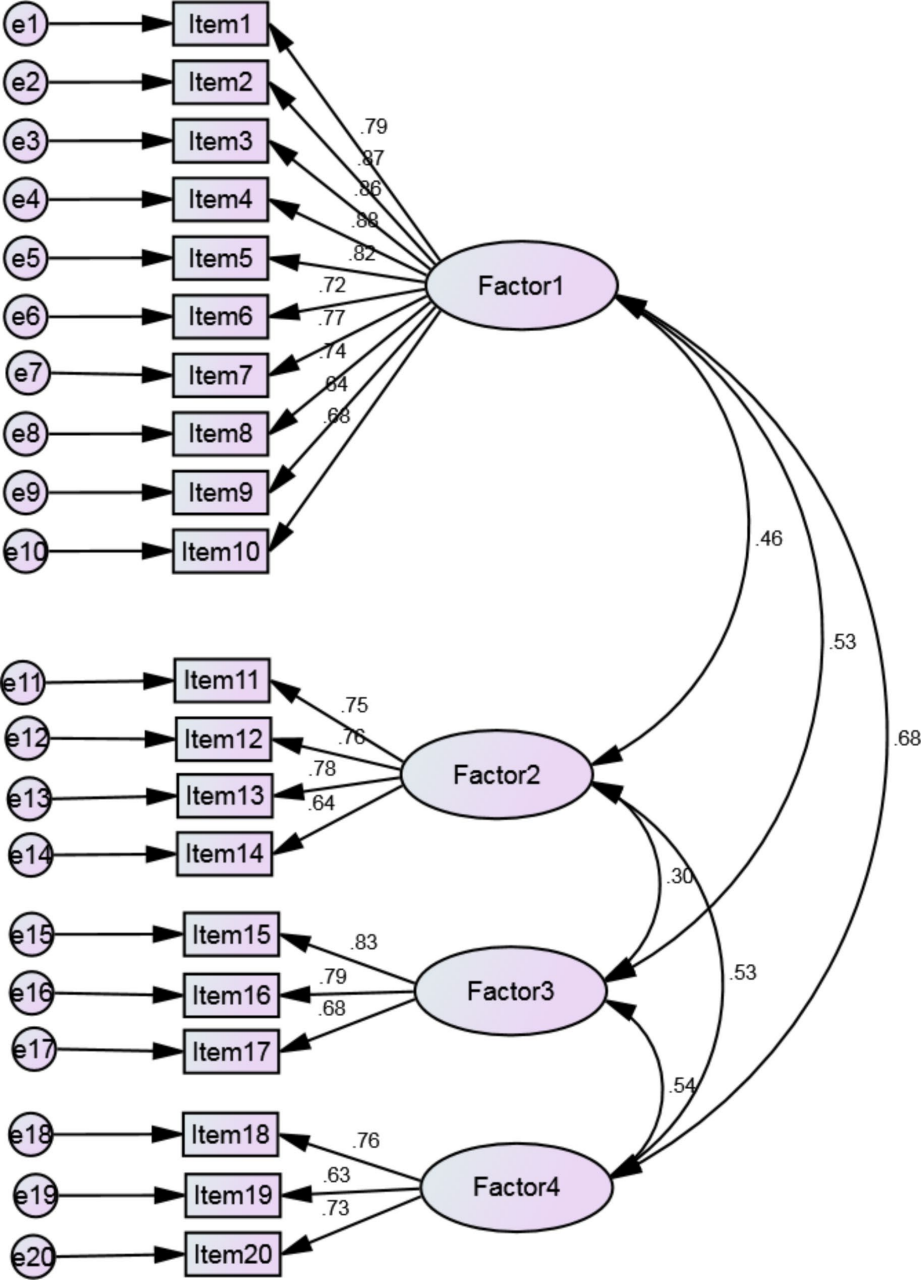
The results of confirmatory factor analysis for the CHFQOLQ-20 (n = 335) Factor 1: Physical functioning; Factor 2: Cognitive functioning; Factor 3: General health; Factor 4: Mental health

The CFA was then performed for the fit of the extracted four-factor model to the data. The results obtained from the CFA eliminated one item from the mental health subscale, and thus, the number of items was reduced to 20. The model showed the following fit indexes for the data: χ^2^/df = 2.214, CFI = 0.947, NFI = 0.91, TLI = 0.937, IFI = 0.947, GFI = 0.899, AGFI = 0.869, RMSEA = 0.063 (95% CI: 0.055–0.071). The results are depicted in Fig. 2. The minimum, maximum, and mean scores of each subscale and the total score of the instrument are presented in Table 3.

**Table 3:** Descriptive statistics for the CHFQOLQ-20 (n = 495) *

	Number of items (possible score)	Minimum	Maximum	Mean (SD)
**Physical functioning**	10 (10–50)	10	48	29.42 (10.34)
**Cognitive functioning**	4 (4–20)	5	20	15.07 (3.64)
**General health**	3 (3–15)	3	15	7.21 (2.59)
**Mental health**	3 (3–15)	3	15	10.62 (2.59)
**Total**	20 (20–100)	20	81	62.41 (15.73)

* Higher scores indicated better quality of life. The total score of the final version range from 20 to 100

### Concurrent validity

As hypothesized, the CHFQOLQ-20 was correlated very well with the following subscales of the SF-36. The detailed correlation coefficients are shown in Table 4.

**Table 4:** Correlation coefficient between the CHFQOLQ-20 and the SF-36 subscales (n = 495)

	PF	RP	BP	GH	VT	SF	RE	MH
**Physical functioning**	0.722**	0.534**	0.638**	0.457**	0.565**	0.588**	0.405**	0.404**
**Cognitive functioning**	0.359**	0.396**	0.364**	0.225**	0.341**	0.397**	0.308**	0.401**
**General health**	0.391**	0.393**	0.470**	0.490**	0.368**	0.388**	0.300**	0.242**
**Mental health**	0.508**	0.425**	0.514**	0.510**	0.605**	0.553**	0.441**	0.627**
**Total**	0.727**	0.595**	0.687**	0.539**	0.636**	0.657**	0.478**	0.528**

PF physical functioning, RP role limitations due to physical problems, BP bodily pain, GH general heal perception, VT vitality, SF social functioning RE role limitations due to emotional problems, MH mental health

* Significant at the 0.05 level

** Significant at the 0.01 level

### Convergent and discriminant validity

As shown in Table 5 the CHFQOLQ-20 was correlated with the following three subscales of the MacNew: physical (r = 0. 772, P < 0.001), emotional (r = 0.671, P < 0.001), and social (r = 0.726, P < 0.001) as expected. In addition, all values for the average variance extracted (AVE), maximum shared squared variance (MSV), and average shared squared variance (ASV) indicated acceptable convergent and discriminant validity. The results are presented in Table 6.

**Table 5:** Correlation coefficient between the CHFQOLQ-20 and the MacNew Heart Disease Quality of Life Questionnaire (n = 495)

	MacNew (Physical)	MacNew (Emotional)	MacNew (Social)
**Physical functioning**	0.741**	0.688**	0.647**
**Cognitive functioning**	0.406**	0.381**	0.38**
**General health**	0.456**	0.487**	0.452**
**Mental health**	0.542**	0.635**	0.675**
**Total**	0.772**	0.671**	0.726**

* Significant at the 0.05 level

** Significant at the 0.01 level

**Table 6:** The results for convergent and discriminant validity (n = 495)

	CR	AVE	MSV	ASV
**Physical functioning**	0.937	0.601	0.473	0.324
**Cognitive functioning**	0.821	0.536	0.282	0.197
**General health**	0.811	0.591	0.292	0.222
**Mental health**	0.752	0.504	0.473	0.349

CR: composite reliability, AVE: average variance extracted, MSV: maximum shared squared variance, ASV: average shared squared variance

### Known-groups comparison

One-way analysis of variance (ANOVA) revealed that the HRQoL scores were significantly different among different functional classes of heart failure (P < 0.05) indicating that the CHFQOLQ-20 could discriminate well between patients who differed in functional class (Table 7).

**Table 7:** The CHFQOLQ-20 scores by the New York Heart Association (NYHA) functional classification (n = 495)

	I	II	III	IV	
	Mean (SD)	Mean (SD)	Mean (SD)	Mean (SD)	P
**Physical functioning**	37.56(8.50)	33.36(9.55)	26.66(10.15)	16.51(9.13)	< 0.0001
**Cognitive functioning**	15.62 (3.24)	15.47 (3.85)	14.89 (3.75)	13.57 (3.70)	0.07
**General health**	8.87 (2.80)	7.8 (2.6)	6. 89 (2.64)	5.75 (2.34)	< 0.0001
**Mental health**	13.25 ( 2.35)	11.10 (3.24)	10.32 (3.37)	7.54 (3.95)	< 0.0001
**Total**	72.34 (12.51)	65.07 (15.49)	56.41 (15.11)	41.60 (12.13)	< 0.0001

### Scaling assumption

The correlation analysis was performed and the results indicated that items belonging to a given factor had higher correlation with its own corresponding factor than other factors lending further support to the structure of the CHFQOLQ-20. The results are shown in Table 8.

**Table 8:** Item-scale correlation matrix for the CHFQOLQ-20 (n = 495)

Items	Factor 1 (Physical functioning)	Factor 2 (Cognitive functioning)	Factor 3 (General health)	Factor 4 (Mental health)
q1	**0.798**	0.362	0.347	0.502
q2	**0.871**	0.352	0.395	0.498
q3	**0.868**	0.331	0.394	0.494
q4	**0.885**	0.360	0.406	0.493
q5	**0.834**	0.301	0.406	0.433
q6	**0.758**	0.273	0.311	0.372
q7	**0.809**	0.414	0.377	0.483
q8	**0.774**	0.401	0.436	0.519
q9	**0.688**	0.415	0.406	0.498
q10	**0.728**	0.335	0.421	0.429
q11	0.400	**0.816**	0.231	0.393
q12	0.284	**0.823**	0.199	0.275
q13	0.326	**0.837**	0.217	0.318
q14	0.392	**0.737**	0.177	0.477
q15	0.416	0.226	**0.844**	0.407
q16	0.415	0.177	**0.877**	0.322
q17	0.404	0.268	**0.805**	0.383
q18	0.564	0.387	0.353	**0.819**
q19	0.447	0.326	0.359	**0.776**
q20	0.421	0.436	0.356	**0.852**

### Reliability

The overall Cronbach’s alpha for the CHFQOLQ-20 was 0.93. It was also calculated separately for each subscale, which was > 0.7, indicating satisfactory results. Also the MacDonald’s Omega showed satisfactory results. The intraclass correlation coefficients (ICC) ranged from 0.7 to 0.92 lending support to the stability of the instrument. (Table 9).

**Table 9:** Reliability measures for the CHFQOLQ-20 (n = 495)

	Cronbach’s alpha	McDonald’s Omega (ω)	ICC (95% CI)
**Physical functioning**	0.939	0.939	0.88 (0.86–0.91)
**Cognitive functioning**	0.817	0.820	0.85 (0.80–0.88)
**General health**	0.803	0.811	0.84 (0.80–0.88)
**Mental health**	0.748	0.757	0.80 (0.76–0.86)
**Total**	0.930	0.931	0.84 (0.80–0.88)

## Discussion

Health-related is an important outcome measure for clinical research. The CHFQOLQ-20 includes important domains of HRQoL as indicated by patients during qualitative stage of the study. Factor analysis extracted four factors of (I) physical functioning, (II) cognitive functioning, (III) general health, and (IV) mental health. These subscales all were confirmed by the CFA.

The first underlying factor identified by factor analysis had a clear reflection of physical functions, and had 10 items. Thus, it was labeled as physical functioning according to ICF. This subscale is the main core of health-related instruments in CHF, and had the maximum percentage of variance in our study. Oldridge et al., in 2014 developed a 14-item questionnaire entitled HeartQoL for ischemic heart failure patients, which had two subscales of physical and emotional. They also included 10 items for the physical domain, similar to CHFQOLQ-20, and the remaining questions (4 items) were related to the emotional domain [38, 39].

Cognitive functioning was the second underlying factor with a lower variance share than the physical domain. Cognitive disorders in HF impair self-care and lead to not reporting the signs and symptoms of disease progression in a timely manner, disability, frequent hospitalizations, decreased QoL, and increased morbidity and mortality, highlighting the significance of assessing the cognitive disorders in patients with HF who are mostly elderly [40]. Despite the significance of the cognitive domain, none of the existing instruments have a cognitive domain, and only the MLHFQ [22] has an item entitled “difficulty in concentration or remembering things” which has been loaded under the emotional domain.

General health was the third underlying factor loaded with three items and mental health which was the fourth underlying factor in CHFQOLQ-20. According to the literature, 30–40% of patients with chronic heart failure experience emotional distress such as depression following impaired physical function, role changes, financial insecurity, and social isolation [40]. Depression in HF patients is correlated with fear due to development of physical symptoms such as dyspnea, and functional limitations. Fear can also lead to denial of disease symptoms and result in not seeking medical attention in time [41].

Similar to HeartQoL [37, 38], CHFQOLQ-20 did not have a social domain. Oldridge et al. explained that the social problems of ischemic heart failure patients may not be unique or strong enough to be suggested as an independent latent construct. On the other hand, the social domain items may be culture- or diagnosis-specific, and since in ischemic heart failure, different diagnoses such as angina, myocardial infarction, and ischemic heart failure are considered, social problems cannot be generalized to the aforementioned three groups of diseases [38, 39]. The present study was conducted on patients with ischemic and non-ischemic HF with different comorbidities, different age groups, and different socioeconomic states, and showed that social problems might not be representative of common concerns for most patients. This also occurred in MLHFQ such that eventually, only two factors of physical and emotional domains were found to be meaningful clusters in MLHFQ [22].

The construct validity and internal consistency of CHFQOLQ-20 indicated that this scale is a promising instrument for assessment of HRQoL in the target population. The first assessment of this new instrument indicated emerging evidence of its validity and reliability. The item-subscales correlation was high, indicating that the items measured the desired concept. With regard to concurrent validity, the correlation between the similar subscales of the CHFQOLQ-20 and the SF-36 was high and significant. Also, the significant correlation of CHFQOLQ-20 with MacNew indicated optimal convergent validity.

Minimum, maximum, and mean subscale and total scores of the CHFQOLQ-20 can be used as a guide in the clinical settings. These results may serve as a unique finding in the clinical setting. Nonetheless, further evaluation of CHFQOLQ-20 is necessary. By offering the norms for each subscale of CHFQOLQ-20, the authors hope that clinicians can make correct decisions in favor of patients in well detectable areas (higher well-being versus lower well-being). For instance, clinicians can refer patients who acquire a low score in mental health domain to a psychologist for psychological counseling. Also, clinicians can optimally rehabilitate patients who have problems in physical functioning domain and acquire a low score in this domain by reenrollment in cardiopulmonary rehabilitation programs or assessment of comorbidities such as sleep apnea and improve their QoL as such. However, longitudinal studies are required to assess the responsiveness and significant clinical changes in the scores. Investigators are recommended to assess the responsiveness of CHFQOLQ-20 to determine its ability to detect significant clinical changes over time. With respect to the low frequency of the missing data, it may be stated that the new questionnaire is relevant and is not too burdensome or difficult to complete. This study did not find a clear pattern for the missing data, which indicates no emergence of systematic missing data.

Finally, it is worth noting that we removed about 17 items from the initial item pool and this might have some implications for an instrument that intends to be patient driven. However, examination of such items revealed that the concept of these items were reflected on other items already included in the current version of the questionnaire.

## Strengths and limitations

One strength of this study was adoption of qualitative and quantitative approaches to develop the instrument based on the experiences of patients living with HF taking into account their cultural background. The participants in this study had different types of HF (ischemic and non-ischemic) and were from different socioeconomic background, which enhances comprehensive assessment of QoL of patients with CHF. A rigorous methodology was another strength of this study, which included the COSMIN checklist and ICF. The CHFQOLQ-20 developed in this study showed satisfactory psychometric properties. The instrument also had practical strengths. First of all, few missing values were observed during the data collection process indicating that it was understandable for patients. Also, since it is a short questionnaire with four unique subscales, its application in clinical studies could be considered as a strength. However, the current study had a number of limitations. Some fit indexes did not meet the acceptable thresholds. Thus, in future studies the CFA indices should be reported again in order to add evidence in this regard. We did not performed responsiveness to detect clinical changes over time. Another limitation was recruitment of patients by convenience sampling. Also, the information belonged to a limited geographical location. Thus, its generalizability to other geographical areas, cultures and races might be examined. Also, this study only evaluated patients attending to HF clinics, and hospitalized patients were not enrolled. Recall bias was another limitation, which might have affected the results since we asked the participants to recall the events of the past month.

## Conclusion

The psychometric properties of the CHFQOLQ-20- were found to be satisfactory. Confirmation of validity and reliability of the CHFQOLQ-20 makes it a unique instrument for use in the future studies on quality of life of patients with chronic heart failure. However, longitudinal studies are required to assess clinically meaningful changes, and responsiveness of the CHFQOLQ-20.

## Data Availability

All data presented in this paper are available from the corresponding authors on reasonable request.

## Abbreviations

CHF: Chronic heart failure
CHFQOLQ-20: 20 items Chronic heart failure quality of life questionnaire
NYHA: New York Heart Association
ICC: Intraclass correlation coefficient
COSMIN: Consensus-based standards for the selection of health measurement instruments
WHO: World Health Organization;
ICF: International Classification of Functioning, Disability and Health
